# Coprophagous Hydrophilid Beetles (Coleoptera, Hydrophilidae, Sphaeridiinae) Distribution in the Polish Carpathians

**DOI:** 10.3390/insects11060355

**Published:** 2020-06-05

**Authors:** Czesław Greń, Andrzej Górz

**Affiliations:** 1 Natural History Department, Upper Silesian Museum in Bytom, pl. Jana III Sobieskiego 2, 41-902 Bytom, Poland; czeslaw.gren@vp.pl; 2 Department of Zoology, Institute of Biology, Pedagogical University of Cracow, Podchorążych 2, 30-084 Krakow, Poland

**Keywords:** coprophagous beetles, Carpathians, Hydrophilidae, Hill numbers, rarefaction, altitudinal distribution, insect migration routes, Poland

## Abstract

Research on coprophagous beetles of the Hydrophilidae family in the Polish Carpathians was conducted from 2011 to 2013. The beetles were caught using baited traps. The research sites were selected to take into account both the horizontal diversity of habitat conditions and the vertical diversity associated with elevation above sea level. During the study, 9589 coprophagous hydrophilid individuals were collected, representing 17 species and five genera. Two species that were new to Poland were found: *Cercyon tatricus* and *Pachysternum capense*. The vertical ranges of the individual species of coprophagous hydrophilid beetles within the Polish Carpathians were determined as well as the elevations above sea level, with the highest and lowest species richness of this group of insects. The capture of *Pachysternum capense* in the Tatra Mountains may indicate the existence of an unrecognized path of migration of small insects from Southern to Northern Europe. The route and mechanisms of their migration are discussed.

## 1. Introduction

The Hydrophilidae family consists mainly of water beetles. Terrestrial species are generally grouped in the highly morphologically and ecologically diverse subfamily, Sphaeridiinae. Most of the Sphaeridiinae species are associated with various kinds of decaying organic substrates (leaf litter, decaying trunks, logs, under rotten bark, garden compost, decaying seaweed, carrion and dung), but there are also genera associated with ants and termites and several of them returned to the aquatic environment. The various types of organic substances inhabited by Sphaeridiinae include mammalian excrement, which is the primary living environment for most European species [1,2,3,4].

Before the present study, Polish coprophagous Hydrophilidae included 22 species, classified into four genera: *Cercyon* (12 species), *Cryptopleurum* (3), *Megasternum* (2) and *Sphaeridium* (5) [5,6].

Owing to research on coprophagous Hydrophilidae both in Poland and all over Europe, this group of beetles is quite well known in the lowlands and highlands of Europe [7,8,9,10,11,12,13,14,15,16,17]. However, there are few data on this group of beetles from the mountainous areas of Europe. This applies to both faunistic data and the altitudinal preferences of individual species [18,19,20,21].

The present study was aimed at acquiring knowledge of the species composition of this group of beetles, their dominance structure and the altitudinal preferences of individual species in the Polish Carpathians.

## 2. Materials and Methods

### 2.1. Study Areas

**Figure 1:**
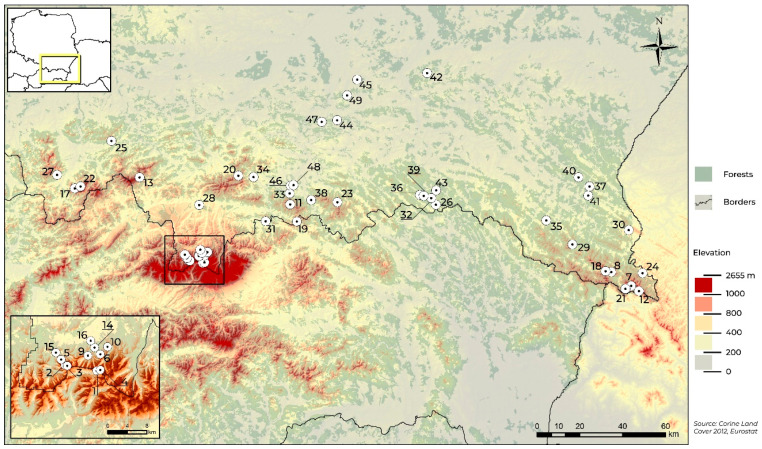
Location of sampling sites in the Polish Carpathians.

The Polish Carpathians as a physical geographical region are a highly diverse natural environment. Therefore, the research sites were selected to take into account both the horizontal diversity of the habitat conditions and the vertical diversity associated with increasing altitude. Beetles were caught at a total of 49 sites (Table 1, Figure 1).

The research on coprophagous hydrophilids was conducted from 2011 to 2013, together with research on the biodiversity of coprophagous Scarabaeoidea of the Polish Carpathians. Each year, the research was begun in the second half of April and continued until the end of October. The exception was the Tatra Mountains where, due to climatic conditions, the research was begun each year in the second half of May and continued until mid-September.

### 2.2. Sampling Method (Collection of Dung Beetles)

Coprophagous beetles were collected using baited traps. These were pitfall traps in the form of plastic containers with a diameter of about 17 cm and a height of 20 cm, filled with about 200 mL of ethylene glycol as a preservative. This part of the trap was buried with its rim level with the ground and covered with 15 mm wire mesh. About 800 g of fresh animal excrement was placed on the mesh. Feces of sheep (about 40%) and cattle (about 60%) were mixed together in order to account for the food preferences of as many species as possible. All traps were placed in open areas with full sunlight, in grassland and herbaceous communities, except for two sites (Przehyba and Przełęcz Krowiarki), where the traps were placed within a subalpine spruce forest (*Plagiothecio-Piceetum tatricum* association), but also in areas without trees (Table 1). Three traps, spaced 10 m apart, were placed at each site. The traps were emptied every 10–12 days [22].

### 2.3. Nomenclature and Systematics

The nomenclature and systematic classification of the Hydrophilidae family was adopted following Przewoźny [23].

Fikáček and Boukal [24] presented a key for the identification of the European genera of the subfamily Sphaeridiinae, including the genus *Pachysternum*, as well as a detailed description of *P. capense*. The following works were used to identify species: [1,14,25,26,27].

### 2.4. Data Analyses

The literature contains many works devoted to the various methods of measuring species diversity [28,29,30]. However, the most coherent means of measuring species diversity was suggested by Hill [31]. Although Hill’s concept was underappreciated for many years, Jost [32,33] has demonstrated that “Hill numbers of order q” are the most coherent method, combining the most commonly used indices into one simple formula. The only element linking the indices used is the exponent q. According to Jost [33], the diversity index based on order q is called “true diversity” [32,33].

In this study, species diversity was determined based on Hill numbers, where:

At q = 0, the abundances of individual species are not taken into account, so the value is simply the species richness of a given area.

At q = 1, we obtain the Shannon diversity index, according to the Hill formula; very abundant and less abundant or rare species all have the same weight, i.e., the value obtained is the most neutral and indicates “true species diversity”. The higher the value at q = 1, the more balanced the dominance structures of the assemblage are.

At q = 2, we obtain an index which is the reverse of Simpson’s index; Hill’s formula gives greater weight to more numerous and common species and less to rare species. Lower values at q = 2 indicate the strong dominance of two or three species in the assemblage.

The diversity profile was calculated using Past 4.02 software.

The diversity profile curves for each vegetation belt were plotted based on the Hill numbers. The three fixed dots on each graph indicate Hill numbers for q = 0, 1 and 2. The slope of the curve reflects the unevenness of the relative species abundances. The more uneven the distribution of relative abundances (i.e., strong dominance of one or two species in the community), the steeper the slope of the curve is [22].

Based on a Monte Carlo null model, the rarefaction method was used to determine the species richness for each interval of elevation above sea level. This method makes it possible to compare sites differing not only in the number of species but also in sample size. Rarefaction curves were calculated and plotted using Past 4.02 software.

## 3. Results

During the study carried out in 2011–2013 in the Polish Carpathians, 9589 coprophagous hydrophilid individuals were collected, representing 17 species and five genera (Table 2). The dominant species in the coprophagous hydrophilid beetle assemblages were *Sphaeridium lunatum*, with a 28.80% share, *Cercyon lateralis* with 11.74% and *Cercyon castaneipennis* with 8.13% (Table 2). According to the dominance scale used, *Cercyon impressus* was a superdominant species in the Polish Carpathians, with 38.46% (Table 2).

On average, seven coprophagous Hydrophilidae species were caught per site. The fewest were caught at Przełęcz Krowiarki (only one species) and the most (14 species) in Ciechania. Sites that were relatively rich in species included Gołkowice Dolne (13 species), Upłaziańska Kopa (12 species), Tarnica, Rozstajne and Brzegi Górne (10 species each) (Table 3). In most cases, the number of species caught at a given site was close to the average which was from five to nine species.

### 3.1. Species Diversity and Dominance Structures of Coprophagous Hydrophilid Beetle Assemblages in the Polish Carpathians

**Figure 2:**
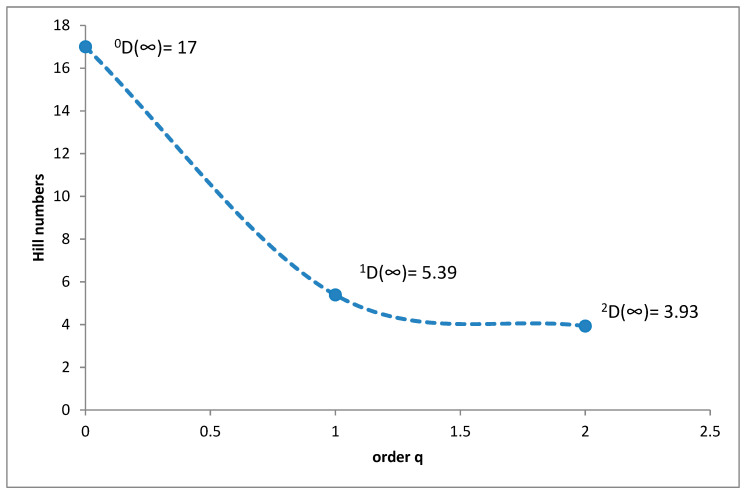
Diversity profile curve plotting Hill numbers for coprophagous hydrophilid beetles in the Polish Carpathians along the elevation gradient.

The species richness of coprophagous hydrophilid species (^0^D) in the Polish Carpathians was 17, ^1^D diversity was 5.39 and ^2^D was 3.93 (Figure 2). *Cercyon impressus* was superdominant throughout the Carpathians, with a share of 38.46%. The group of dominants comprised *Sphaeridium lunatum*, with a 28.80% share, *Cercyon lateralis*, with 11.74% and *Cercyon castaneipennis* with 8.13% (Table 2).

**Figure 3:**
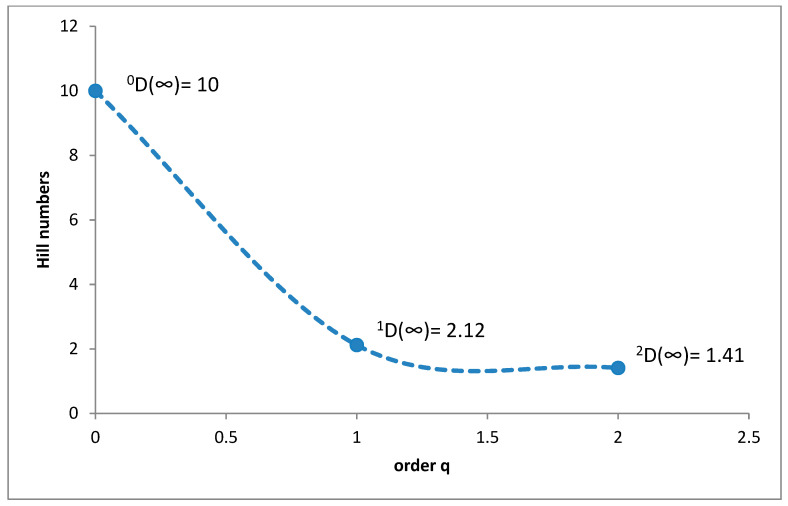
Diversity profile curve plotting Hill numbers for the alpine belt.

**Figure 4:**
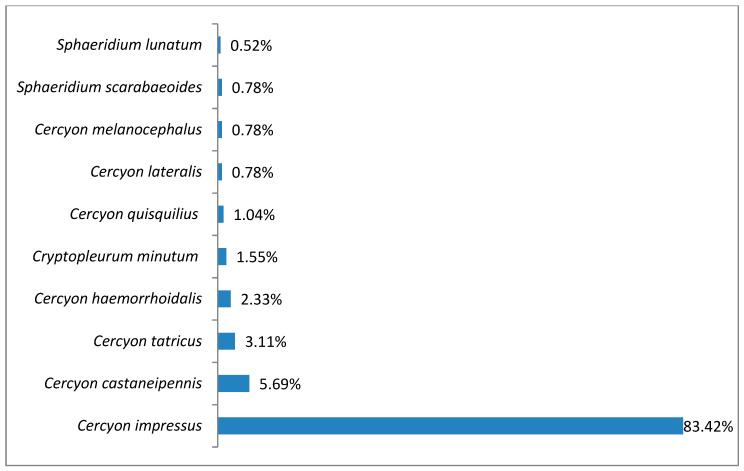
Percentage shares of all coprophagous hydrophilid beetle species recorded in 2011–2013 in the alpine belt.

In the alpine belt, species richness of coprophagous hydrophilids (^0^D) was 10, ^1^D diversity was 2.12, and ^2^D was 1.41 (Figure 3). *Cercyon impressus* was a superdominant in this region, with a share of 83.42%, while the dominant was *Cercyon castaneipennis* with 5.69%, (Figure 4).

**Figure 5:**
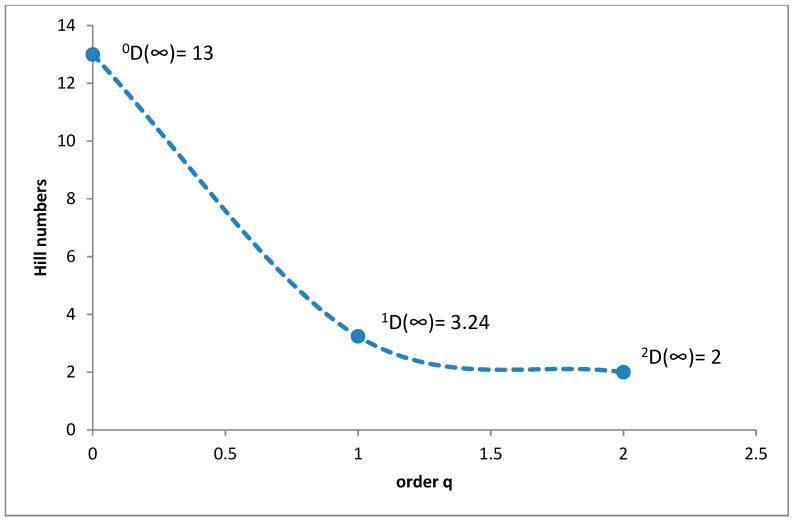
Diversity profile curve plotting Hill numbers for the subalpine belt.

**Figure 6:**
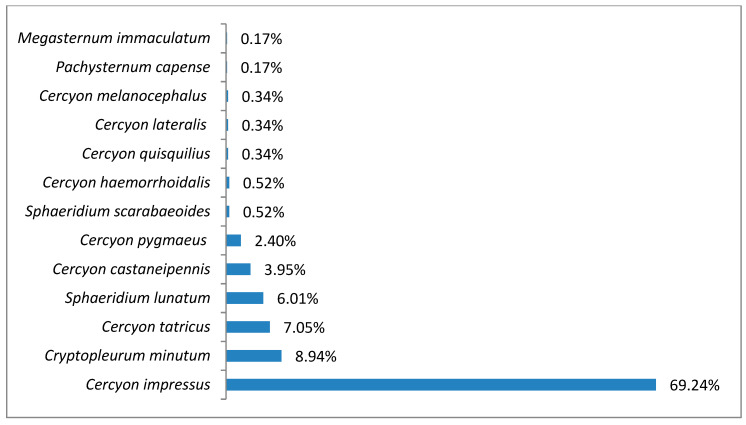
Percentage shares of all coprophagous hydrophilid beetle species recorded in 2011–2013 in the subalpine belt.

In the subalpine belt, the species richness was 13 species (^0^D = 13), while diversity at the ^1^D level was 3.24 and ^2^D was 2 (Figure 5). The superdominant in this belt was *Cercyon impressus* (69.24%), and three species were dominants (Figure 6).

**Figure 7:**
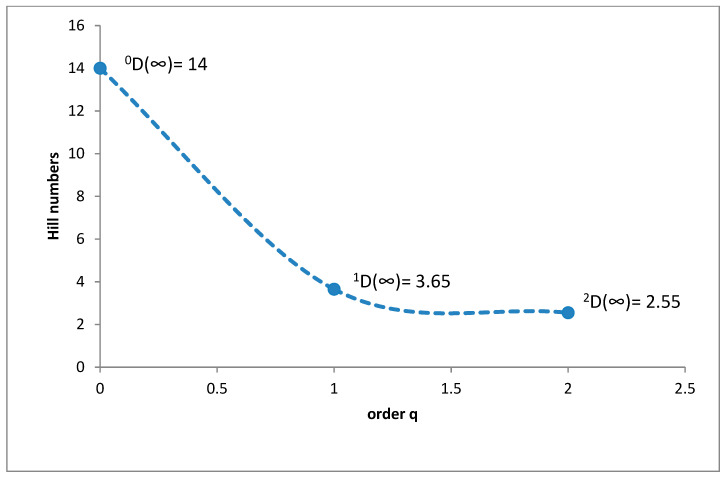
Diversity profile curve plotting Hill numbers for the upper montane belt.

**Figure 8:**
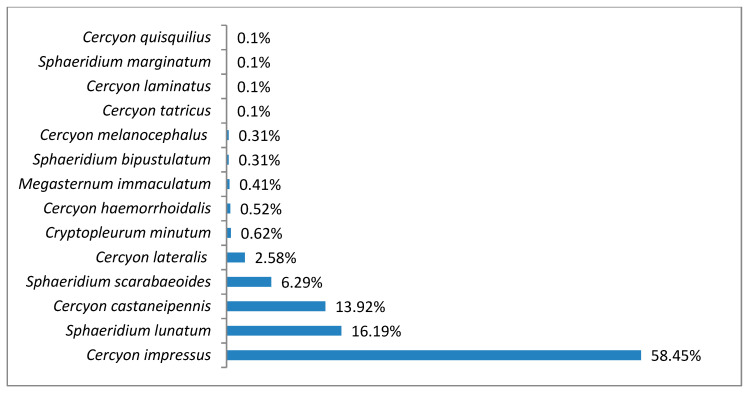
Percentage shares of all coprophagous hydrophilid beetle species recorded in 2011–2013 in the upper montane belt.

In the upper montane belt, the species richness was 14 species (^0^D = 14), while diversity at the ^1^D level was 3.65 and ^2^D was 2.55 (Figure 7). *Cercyon impressus* was a superdominant in this region, with a share of 58.45%, and three species were dominants (Figure 8).

**Figure 9:**
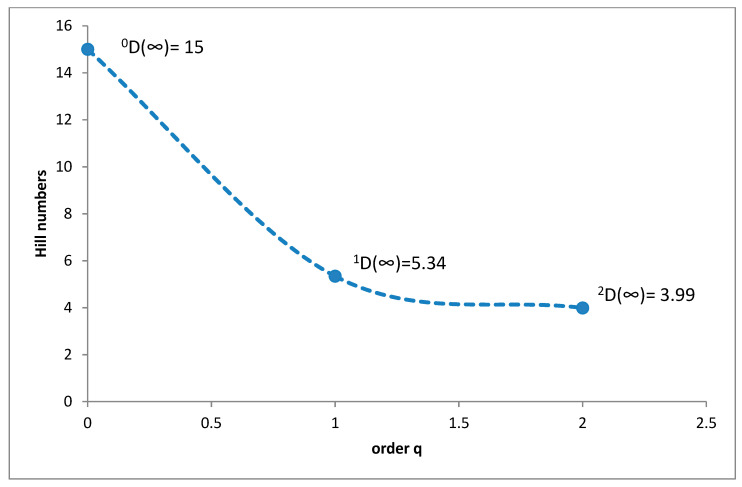
Diversity profile curve plotting Hill numbers for the lower montane belt.

**Figure 10:**
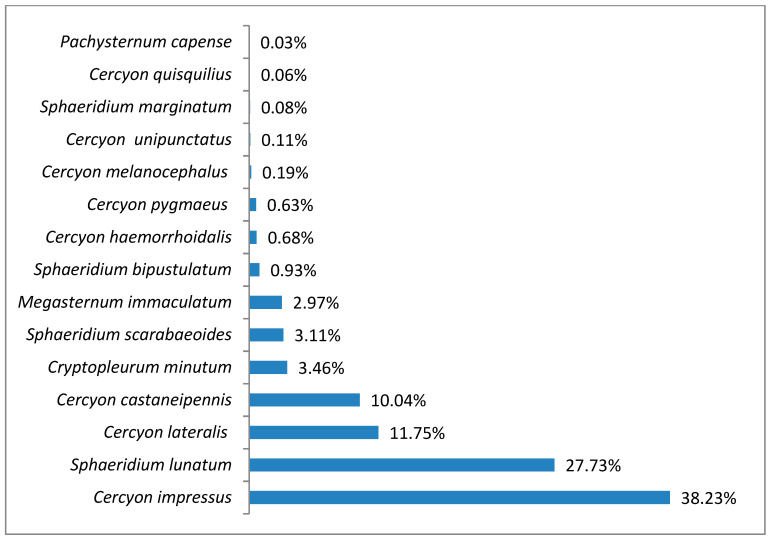
Percentage shares of all coprophagous hydrophilid beetle species recorded in 2011–2013 in the lower montane belt.

The greatest species richness was noted in the lower montane belt. There were 15 coprophagous hydrophilid species found here (^0^D), with a ^1^D diversity of 5.34 and ^2^D = 3.99 (Figure 9). *Cercyon impressus* was a superdominant in this region, with a share of 38.23%, while the dominants were *Sphaeridium lunatum* with 27.72%, *Cercyon lateralis* with 11.75% and *Cercyon castaneipennis* with 10.04% (Figure 10).

**Figure 11:**
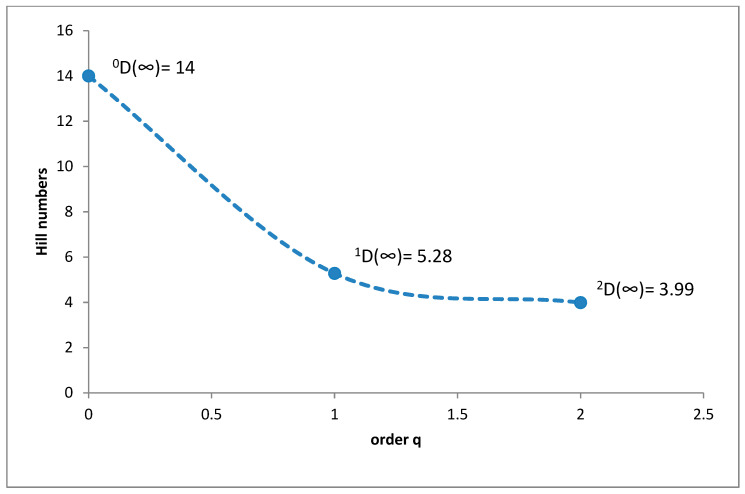
Diversity profile curve plotting Hill numbers for the foothills belt.

**Figure 12:**
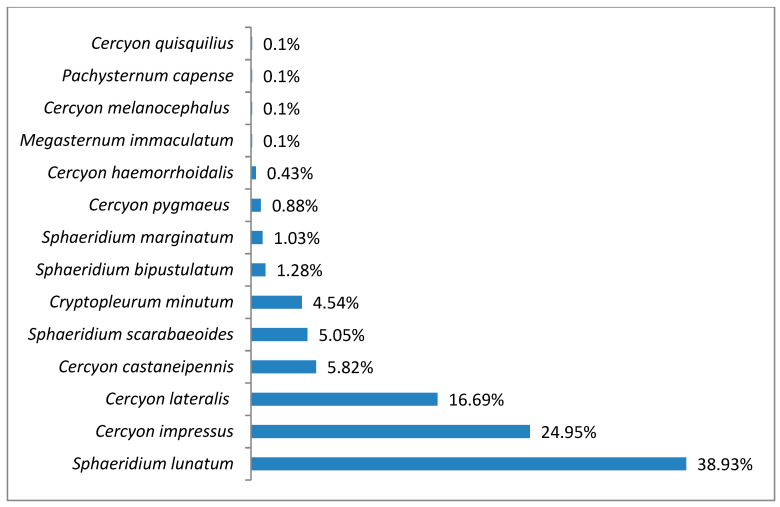
Percentage shares of all coprophagous hydrophilid beetles species recorded in 2011–2013 in the foothills belt.

In the foothills belt, the species richness was 14 species (^0^D = 14), while diversity at the ^1^D level was 5.28 and ^2^D was 3.99 (Figure 11). The superdominant in this belt was *Sphaeridium lunatum* (38.93%), while the dominants were *Cercyon impressus* with 24.95%, *Cercyon lateralis* with 16.69%, *Cercyon castaneipennis* with 5.82% and *Sphaeridium scarabaeoides* with 5.05% (Figure 12).

### 3.2. Altitudinal Distribution of Species in the Polish Carpathians

**Figure 13:**
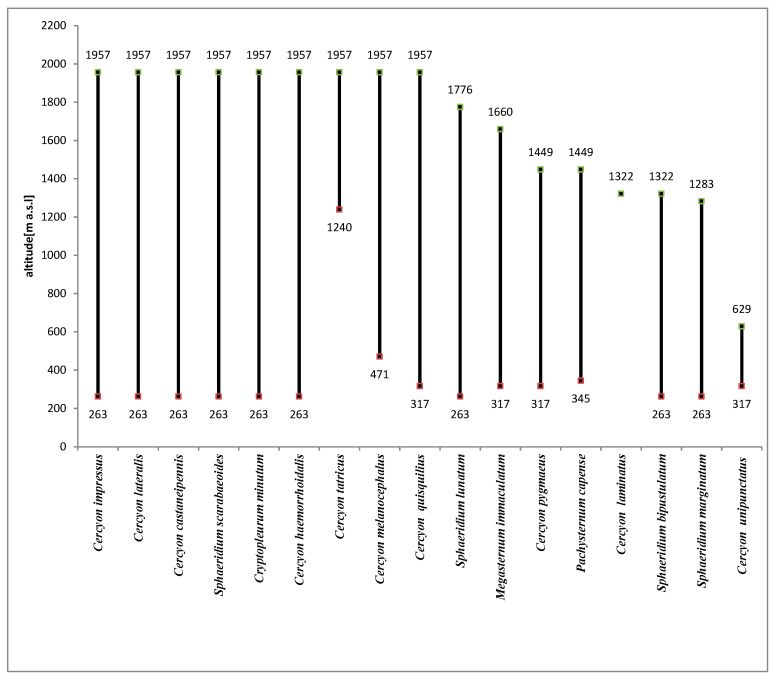
Vertical range of coprophagous hydrophilids (Coleoptera: Hydrophilidae) in the Polish Carpathians.

The species richness of all the vegetation/climate belts within the Polish Carpathians, except for the alpine belt, was very similar (Figure 13). This indicates that the coprophagous Hydrophilidae species found here have a high tolerance to climatic and environmental conditions. The alpine belt, despite extremely unfavourable climatic conditions, is also inhabited by a relatively large group of species (Figure 3). Compared to the lower montane range, with the highest species richness (15 species from this group), the alpine belt cannot be described as especially poor. This indicates that coprophagous Hydrophilidae are not highly dependent on climatic and environmental conditions but only on food substrate availability.

### 3.3. Overview of Collected Species

*Cercyon (Cercyon) castaneipennis* (Vorst, 2009)

This recently described species has been recorded in Belarus, the Canary Islands, Russia, the Czech Republic, Latvia, the Netherlands, Poland, Slovakia and Sweden [34]. In the past, it was not distinguished from *C. obsoletus* (Gyllenhal, 1808), also known in Poland, although it was described as the color aberration *C. obsoletus* ab. *rubridorsis* by Reitter in the early twentieth century [35]. For this reason, its distribution in Poland is not yet well known. Some of the old data on *C. obsoletus* undoubtedly refer to this species. It has already been recorded in ten regions: the Baltic Coast, the Masurian Lake District, the Wielkopolska-Kujawska Lowland, the Mazovian Lowland, Białowieża Forest, Upper Silesia, the Kraków-Wieluń Upland, the Malopolska Upland, the Western Beskids and the Bieszczady Mountains [6,14,15,36,37]. It lives in the excrement of large herbivores (cows, horses and others) in diverse habitats [13].

**Figure 14:**
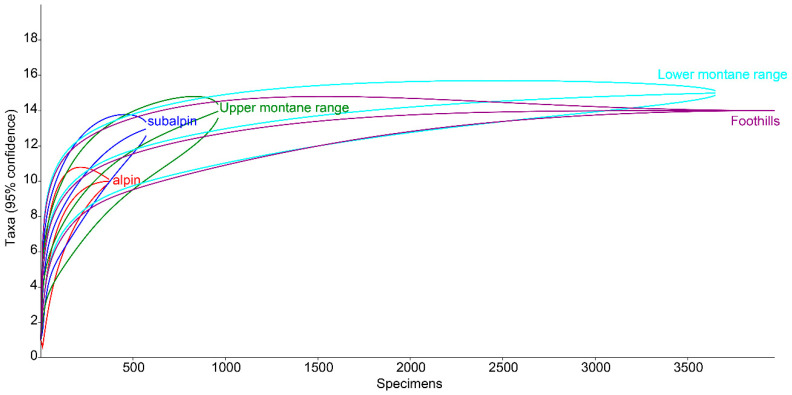
Rarefaction curves for species richness of coprophagous hydrophilid beetles in the Polish Carpathians along the elevation gradient.

Recorded at 36 sites in the Polish Carpathians (Table 3), it was found at all altitude gradients in the study area (Figure 14). Within the upper montane range and alpine belt, it belonged to the group of dominants (Figure 4 and Figure 8).

*Cercyon (Cercyon) haemorrhoidalis* (Fabricius, 1775)

This very widely distributed Palearctic species was also introduced to the Australian, Oriental, Nearctic and Neotropical regions [34,38]. It inhabits the feces of various herbivorous mammals as well as rotting plant debris, compost piles and carrion. It has also been found in the nests of birds and small rodents [1,38,39,40]. In Poland, it is widespread throughout the country, frequent and in places quite abundant. In the Polish Carpathians, it has been recorded in the Western Beskids, Eastern Beskids and the Bieszczady Mountains [41,42,43,44].

It was found at 17 sites in the Polish Carpathians (Table 3). Its vertical range reaches the alpine belt, i.e., a minimum of about 2000 m a.s.l. It is new to the Tatra Mountains.

*Cercyon (Cercyon) impressus* (Sturm, 1807)

This European species was introduced to North America [34]. It lives in all types of decaying plant and animal remains but prefers the excrement of herbivores, especially even-toed ungulates [40]. It is widespread throughout Poland. In the Polish Carpathians, it has been recorded in the Western Beskids, Eastern Beskids, Bieszczady Mountains and Tatra Mountains [36,41,42,43,44,45,46].

Found at 45 sites in the Polish Carpathians (Table 3), it is the most abundant species of all those recorded (Table 2). Only within the foothills was it a dominant species (Figure 12), while in all other vegetation/climate belts it was a superdominant (Figure 4, Figure 6, Figure 8 and Figure 10). It was found at all altitude gradients in the study area (Figure 14).

*Cercyon (Cercyon) lateralis* (Marsham, 1802)

This Palearctic species isvery widely distributed in nearly all of Europe and in the Russian part of Asia, as far as Kazakhstan and the Russian Far East. It was introduced to North America, where it became fully acclimated [34,47]. It is found in the excrement of horses, cows, European bison, deer and many other mammals, in rotting plant debris and in rotting fungi. It has also been found in the nests of birds: *Turdus philomelos* (C. L. Brehm) and *Pernis apivorus* (Linnaeus) [40,47]. It is widely distributed throughout Poland, where it is a common and abundant species. In the Polish Carpathians, it has been recorded in the Western Beskids, Eastern Beskids, Bieszczady Mountains and Tatra Mountains [36,41,42,43,44,45,46,48].

Found at 42 sites in the Polish Carpathians (Table 3), it was found at all altitude gradients in the study area. Within the foothills and lower montane range, it belonged to the group of dominants (Figure 10 and Figure 12).

*Cercyon (Cercyon) melanocephalus* (Linnaeus, 1758)

This species with Palearctic range is widely distributed in Europe and has been found in Asia in Lebanon, Russia (Western and Eastern Siberia) and Uzbekistan [34,49]. It lives in the excrement of herbivores, mainly even-toed ungulates [40]. In Poland, it is distributed throughout the country, but it is seen rarely and only as isolated specimens. It is known across the entire arc of the Polish Carpathians except the Pieniny Mountains [36,41,42,43,44,45,46].

It was found at nine sites in the Polish Carpathians (Table 3). Its vertical range reaches the alpine belt, i.e., a minimum of about 2000 m a.s.l. (Figure 14).

*Cercyon (Cercyon) pygmaeus* (Illiger, 1801)

This is a widely distributed Palearctic species, reaching Eastern Siberia. It has been introduced to North America [34]. It mainly inhabits the feces of herbivores, most often even-toed ungulates [40]. In Poland, it is widespread throughout the country. It is one of the most common coprophagous representatives of the genus. In the Polish Carpathians, it has been recorded in the Western Beskids, Eastern Beskids and Bieszczady Mountains [41,42,43,44,45,46].

Found at 12 sites in the Polish Carpathians (Table 3), its vertical range reaches up to about 1500 m a.s.l. (Figure 14).

*Cercyon (Cercyon) quisquilius* (Linnaeus, 1761)

This species is widespread throughout the Palearctic region. It has been introduced to North America, South America and Australia [34,38,50]. It is polysaprophagous, inhabiting mainly the excrement of mammals (cows, horses, sheep and others). It is often found in compost, rotting fungi and plant waste [39,40,47,49,51,52,53]. It has also been caught in the nest of a black stork, *Ciconia nigra* (Linnaeus, 1758) [54]. It is widespread throughout Poland and has been recorded many times across the entire Polish Carpathians, except for the Tatra Mountains [36,41,42,43,44,55].

Found at seven sites in the Polish Carpathians (Table 3), its vertical range reaches the alpine belt, i.e., a minimum of about 2000 m a.s.l. (Figure 14). It is new to the Tatra Mountains.

*Cercyon (Cercyon) tatricus* (Endródy–Younga 1967)

This is a mountain species with a very interesting distribution. Until now, it had been recorded in two areas separated by a great distance—the Carpathians in Europe (Slovakia, Ukraine and Romania) and the Russian Far East (Amur Oblast, Khabarovsk Krai, Primorsky Krai and Kamchatka) [27,34]. It is found in the feces of even-toed ungulates, as well as that of bears [27,40]. It has been recorded in the High Tatras in Slovakia—the closest location to Poland—as well as the Belianske and Low Tatras [40]. It is also known in the Ivano-Frankivsk Oblast of Ukraine [27].

A new species for Polish fauna, it was found at only five sites in the Tatras (Table 3), in the subalpine and alpine belts (Figure 14). Within the subalpine belt, it was one of the dominant species (Figure 6).

*Cercyon (Cercyon) unipunctatus* (Linnaeus, 1758)

This is a widespread Palearctic species found all over Europe, except for its southernmost parts. In Asia, it is known in Kazakhstan, Russia, Mongolia, northern parts of China and Northern Japan [34,50]. In the mid-nineteenth century, it was introduced to North America, where it also spread rapidly [39]. It inhabits various types of decaying plant remains, mammal excrement (especially that of cows, horses and sheep) and chicken manure. It is sometimes found in decaying plant debris near water and in bird nests [54,56]. In Poland, it is widespread throughout the country, where it is very common and numerous. In the Polish Carpathians, it has been recorded many times in the Western Beskids, Eastern Beskids and the Bieszczady and Pieniny Mountains [41,43,44,55,57,58].

Only four individuals were found in the Polish Carpathians (Table 2), at three sites (Table 3). It was caught at elevations from 317 m to 629 m a.s.l. (Figure 14).

*Cercyon (Paracycreon) laminatus* (Sharp, 1873)

This species was originally widespread in the Eastern Palearctic (Japan, Russian Far East and China). It was introduced to Europe in the mid-twentieth century, where it became fully acclimated [40]. It has now been found in Hawaii, Taiwan, Australia and Chile [38,59]. It lives in various kinds of decaying organic matter and the excrement of various mammals [40]. It is often encountered in Poland. Although it was first recorded in Poland relatively recently, it has already been found in 14 regions. In the Polish Carpathians, it is known in the Eastern Beskids and the Bieszczady and Pieniny Mountains [55,60,61,62].

Only one individual was found in the Polish Carpathians (Table 2), on Tarnica in the Bieszczady Mountains (1322 m a.s.l.).

*Pachysternum capense* (Mulsant, 1844)

This is a species originating in sub-Saharan Africa, from which it was introduced to Northern Africa, North America, South America, Australia, Europe and many islands including the Canary Islands, Madeira, the Comoros and Mauritius [63]. In Europe, it was first recorded on the basis of specimens caught in Greece in 1997 [24]. It has spread significantly since then and is currently known in France, Italy, Hungary and Romania [34,63,64]. This shows that this African species is fully acclimated in Europe and is rapidly expanding its range.

A new species for Polish fauna. In the Polish Carpathians, six individuals were caught at five sites: Ciechań (629 m a.s.l.), Rozstajne (452 m a.s.l.), Stasiówka (395 m a.s.l.) and Zawada (384 m a.s.l.) in the Eastern Beskids and Upłaziańska Kopa (1449 m a.s.l.) in the Tatras (Table 3). The nearest known sites of this species in Romania and Hungary are more than 350 km away [63].

*Megasternum immaculatum* (Stephens, 1829)

*M. immaculatum*, although described by Stephens almost 200 years ago, was until recently treated as a synonym of *M. concinnum* (Marsham, 1802) [65]. It was not restored to the status of a separate species until a few years ago [66]. Diagnostic characters provided by the authors, based on the structure of the male copulation apparatus and the upper body color, enable some distinction between the two species. Therefore, previously published data on the occurrence of *M. concinnum* require verification, since in many cases they probably refer to *M. immaculatum*. To date, it has been confirmed in the United Kingdom [6,34], Poland [6], Western and Eastern Siberia [67] and Bulgaria [68].

Although the authors of the Catalogue of Palaearctic Coleoptera [34] explain in the comments that further research is underway to clarify the taxonomic questions regarding the genus *Megasternum* (associated with numerous synonyms attributed to *M. concinnum*), they do not question the presence of two distinct species in Central Europe.

The species was found at 12 sites in the Polish Carpathians (Table 3). Its vertical range reached up to about 1660 m a.s.l.

*Cryptopleurum minutum* (Fabricius, 1775)

This is a widely distributed Palearctic species, absent from North Africa, which was introduced to North America [34]. It lives mainly in animal excrement and is also often found in decaying plant debris [40]. It is common throughout Poland. It has been recorded many times over the entire arc of the Polish Carpathians [36,41,42,43,44].

It was found at 28 sites in the Polish Carpathians (Table 3). Its vertical range reached up to a minimum of about 2000 m a.s.l. (Figure 14).

*Sphaeridium lunatum* (Fabricius, 1792)

This is a widely distributed Palearctic species that was introduced to North America [34]. It lives in the excrement of various herbivores, mainly even-toed ungulates [40]. It is common in Poland and probably distributed throughout the country. In the Polish Carpathians, it was previously recorded in the Western Beskids [69], Eastern Beskids [70] and the Bieszczady Mountains [43,58].

It was caught at 38 sites in the Polish Carpathians (Table 3). It is the most numerous species of the genus and the second most numerous of all recorded representatives of Hydrophilidae (Table 2). It was a superdominant in the foothills (Figure 12), while within the lower montane range, upper montane range and subalpine belt, it belonged to the group of dominants (Figure 6, Figure 8 and Figure 10). Its vertical range reached up to 1800 m a.s.l. (Figure 14). It is new to the Tatras.

*Sphaeridium scarabaeoides* (Linnaeus, 1758)

This is a Palearctic species that was introduced to Africa, Australia and North America [27]. It lives in the excrement of various herbivores, mainly even-toed ungulates [35]. In Poland, it is common and frequently encountered everywhere. In the Polish Carpathians, it had previously been recorded in the Western Beskids, Eastern Beskids and the Bieszczady and Tatra Mountains [36,38,39,53].

It was caught at 35 sites in the Polish Carpathians (Table 3), at all altitude gradients within the study area (Figure 14).

*Sphaeridium bipustulatum* (Fabricius, 1781)

This is a widely distributed Palearctic species that was introduced to North America [34]. It lives in the excrement of various herbivores, mainly even-toed ungulates [40]. In Poland, it is common and frequently encountered. In the Polish Carpathians, it had previously been recorded in the Western Beskids, Eastern Beskids and the Bieszczady and Tatra Mountains [41,43,44,58]. It was caught at 15 sites within the Polish Carpathians (Table 3). Its vertical range reached up to 1322 m a.s.l. (Figure 14).

*Sphaeridium marginatum* (Fabricius, 1787)

This is a widely distributed Palearctic species, also introduced to North America [34]. Like other European representatives of this genus, it lives in the excrements of various herbivores, mainly even-toed ungulates [40]. For a long time, it was treated as a variant of *S. bipustulatum*, until Van Berge–Henegouwen [26] demonstrated that it was a separate species. For this reason, its distribution in Poland is not yet well known. It has been recorded in five regions: the Wielkopolska-Kujawska Lowland [5,15,37,71,72,73], Podlasie [74], the Białowieża forest [75], Upper Silesia [6,36] and the Malopolska Upland [76,77].

Not previously recorded in the Polish Carpathians, it was found at 12 sites (Table 3). Its vertical range reaches up to about 1283 m a.s.l. (Figure 14).

## 4. Discussion

**Figure 15:**
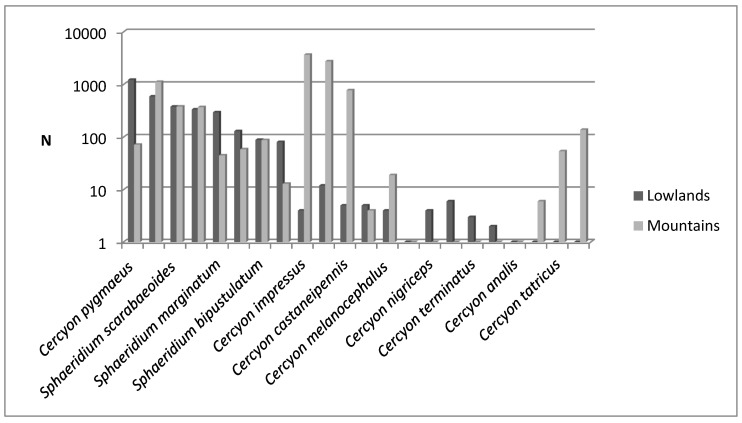
Comparison of abundance of species found in the mountains (Polish Carpathians) and lowlands (Wielkopolska Lowland—based on Przewoźny, Bajerlein) [15].

Research on the coprophagous Hydrophilidae of the Polish Carpathians was conducted from 2011 to 2013, during which time 17 species belonging to five genera were found (Table 2). The species *Pachysternum capense* and *Cercyon tatricus* are new to Polish fauna, so the number of coprophagous Hydrophilidae species recorded in Poland has increased to 24. A characteristic feature of coprophagous communities of the beetles of the Hydrophilidae family is usually the dominance of species of the genus *Cercyon* [8,15]. This was confirmed by the results of the study, as of the 17 species found, 10 belong to the genus *Cercyon*, and the superdominant within the Polish Carpathians was *Cercyon impressus* (Table 2). The only typical mountain species was *Cercyon tatricus*, which in Poland is found only within the Tatra Mountains (Table 2). However, a comparison of the abundance of species common to the Carpathians and the Wielkopolska Lowland [15] indicates that *Cercyon impressus*, *C. castaneipennis* and *Spheridium lunatum* prefer a mountain environment (Figure 15). Species of the genus *Megasternum* were grouped together on the graph, without distinguishing the species, because *M. concinnum* was not separated into two species [66] until after the research that was conducted in Wielkopolska Lowland [15], and it is not currently possible to determine which species these data refer to.

As in the present research, a study of coprophagous beetles that was conducted on two mountain pastures (about 600 and 800 m a.s.l.) in the Southern Czech Republic [21] found that the most numerous species representing the Hydrophilidae family were *Cercyon impressus*, *Sphaeridium lunatum*, *Cercyon lateralis*, *C. castaneipennis* and *Spaheridium scarabaeoides*.

It is a generally accepted rule that in well-researched communities, species with very large populations are the fewest, but it is to these species that most individuals in the community belong [78]. This rule is confirmed by the results of this study: both within the Polish Carpathians as a whole and in individual vegetation/climate belts, there were few highly abundant species, which constituted the majority of the community, and many less numerous species (Table 2, Figure 4, Figure 6, Figure 8, Figure 10 and Figure 12). Therefore, the species diversity of coprophagous hydrophilid beetles in the Polish Carpathians as expressed by the Hill numbers is not very high (^1^D = 5.39 and ^2^D = 3.93; Figure 2). The lowest species diversity, expressed in Hill numbers (q order), was found within the alpine belt (Figure 3) and subalpine belt (Figure 5), while the greatest diversity, expressed in Hill numbers, occurs within the lower montane range (Figure 9) and the foothills (Figure 11).

**Figure 16:**
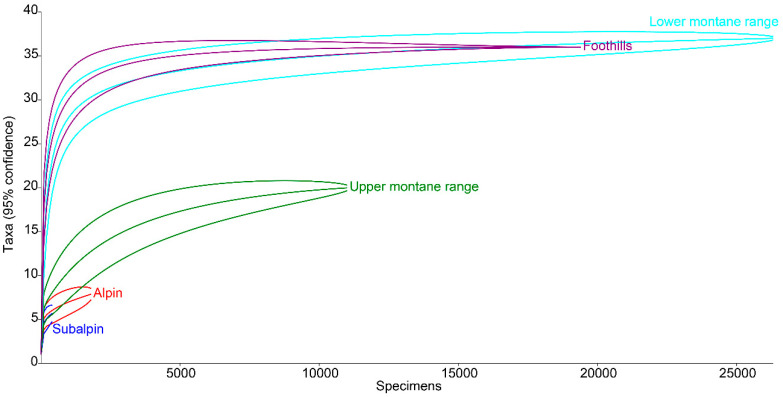
Rarefaction curves for species richness of dung beetles (Scarabaeoidea) in the Polish Carpathians along the elevation gradient [22].

In terms of vertical range, the greatest species richness is usually found at medium elevations [79,80]. Research has shown that in the case of the Polish Carpathians, this refers to habitats located in the range of 400–700 m a.s.l., i.e., within the lower montane belt and foothills [22]. As in the present study on coprophagous Hydrophilidae, the most coprophagous species of the superfamily Scarabaeoidea were also found within this range (Figure 16), with the fewest found in the subalpine and alpine belts. However, this graph shows that Carpathian dung beetle assemblages include species with different habitat and climate requirements [22].

It should be noted that coprophagous hydrophilid beetles were caught in the Polish Carpathians by the same method and at the same sites as dung beetles (Scarabaeoidea).

Thus, a comparison of both sets of rarefaction curves (Figure 13 and Figure 16) shows that the Carpathian assemblages of coprophagous hydrophilid beetles consist mainly of ubiquitous species, i.e., those with high ecological plasticity. This is also confirmed by the analysis of the vertical ranges of coprophagous hydrophilids (Figure 14).

Among the species recorded, *Cercyon impressus*, *C. lateralis*, *C. castaneipennis*, *C. haemorrhoidalis*, *Cryptopleurum minutum* and *Spheridium scarabaeoides* were present at all altitude gradients in the study area (Figure 2). The species *Cercyon melanocephalus*, *C. quisquilius* and *Spheridium lunatum* were also found at nearly all altitude gradients (Figure 14).

In the case of *Cercyom laminatus* and *C. unipunctatus*, however, it was impossible to determine the true vertical range due to the small number of specimens and the small number of sites where they were caught.

It is worth noting that within the Polish Carpathians, the site that was richest in species was the one in Ciechania, where 14 coprophagous hydrophilid beetles were found during the study (Table 3; Figure 1). It should be added that the most coprophagous Scarabaeoidea species—32 species of dung beetles—were also caught at this site [22].

Ciechania is located within the Low Beskid Mountains, which is an exceptional area in terms of both habitat (with many warm, sunny, open pastures) and food availability for coprophagous beetles. Extensive farming, including a large share of cow, sheep and horse farming, is conducted in this area. In addition, there are about 1000 deer in the Low Beskids [22].

It is due to all of these factors—the presence of optimal habitats and adequate food availability throughout the growing season—that this area has the greatest species richness of coprophagous Hydrophilidea and Scarabaeoidea in the entire Polish Carpathians.

The fact that *Pachysternum capense* was caught in the Tatras at 1445 m above sea level seems to be an extremely interesting phenomenon. This is a species originating in sub-Saharan Africa which, according to research, has acclimated in Europe and is rapidly expanding its range [24,34,63,64]. However, until now this species has inhabited areas with a much milder climate than that prevailing in Poland, especially in the Tatra Mountains. The other sites where this species that is new to Polish fauna was caught are unsurprising, as they are in the vicinity of the Dukla Pass (Ciechań and Rozstajne), the main migration route for fauna and flora across the Carpathians from Southern Europe [81]. Could the capture of one individual in the Tatras be accidental?

Atmospheric transport is well known to be the dominant means of migration for small insects. A great number of small species of insects are most likely adapted to flight during the day under convective conditions, exploiting upward and horizontal air currents to disperse beyond their natal sites [82].

The Tatra Mountains, due to their height, undoubtedly constitute a migration barrier for most animals. They have thus far not been considered a potential migration corridor from Southern to Northern Europe, especially for insects.

However, one of the characteristic features of the Tatra climate is the occurrence of local winds, including the “Liptov” wind [83]. This arises due to stronger heating of the southern slopes of the Tatra Mountains and the Liptov Basin relative to the northern slopes and valleys. In consequence, strong convective air movements occur on the southern side and flow over the ridge of the pass towards the northern slopes [83]. *Pachysternum capense* was caught in the part of the Tatra Mountains where the Liptov wind occurs. It should be added that *Bodilopsis rufa* (Moll, 1782) was also found. It is not without significance that the center of the occurrence of *B. rufa* in the Polish Carpathians was at sites between 400 and 600 m a.s.l. This species was caught only in open areas. Single individuals were caught in the Tatra Mountains at about 1800 m a.s.l. [22].

Both species were caught as single specimens. It can therefore be assumed that they were transported over the pass by the Liptov wind and then caught on the northern side of the Tatras. They are certainly not the only insect species that can migrate from the south to the north of the Tatras by this route. This is confirmed by the high activity of bats at the passes where the Liptov wind phenomenon is observed (unpublished data from Krzysztof Piksa). Insects are well known to be the only diet of bats found in this part of Europe [84,85].

Hence, the Western Tatras, where the Liptov wind occurs, are likely to be a migration corridor for small flying insects.

## 5. Conclusions

In our study, we found 17 coprophagous Hydrophilidae species from the subfamily Sphaeridiinae in the Polish Carpathians. Two of them, *Cercyon tatricus* and *Pachysternum capense*, are species new to Polish fauna. Species of the genus *Cercyon* were primarily dominant in the Polish Carpathians, and *Cercyon impressus* was a superdominant.

The species richness of this group of beetles was highly similar in all the vegetation and climate belts studied within the Polish Carpathians, except for the Alpine belt.

The Carpathian assemblages of coprophagous hydrophilid beetles can be said to consist mainly of ubiquitous species, i.e. species with high ecological plasticity.

The fact that *Pachysternum capense* was caught in the Tatras at an altitude of 1445 m a.s.l. may indicate that there is an unrecognized path of migration of small insects from southern to northern Europe through the Western Carpathians.

## Figures and Tables

**Table 1:** List of sites of research on coprophagous hydrophilids in the Polish Carpathians.

No. on Map	Site	Elevation (m a.s.l.)	Geographical Coordinate System in DD (Decimal Degrees)	Vegetation Belt	Type of Plant Association
1	Kasprowy Wierch	1957	N 49.22850° E 019.98712°	Alpine	*Trifido-Distichetum*
2	Twarda Galeria	1776	N 49.23967° E 019.89746°	Alpine	*Trifido-Distichetum*
3	Kocioł Mułowy	1707	N 49.23848° E 019.90410°	Alpine	*Trifido-Distichetum*
4	Hala Gąsienicowa	1660	N 49.23026° E 019.99769°	Subalpine	*Trifido-Distichetum*
5	Upłaziańska Kopa	1449	N 49.25033° E 019.88700°	Subalpine	*Gladiolo-Agrostietum*
6	Skupniów Upłaz	1390	N 49.25930° E 019.99849°	Upper montane range	*Gladiolo-Agrostietum*
7	Tarnica	1322	N 49.07629° E 022.72537°	Subalpine meadows (Poloninas)	*Poo-Deschampsietum*
8	Połonina Caryńska	1283	N 49.13834° E 022.60265°	Subalpine meadows (Poloninas)	*Poo-Deschampsietum*
9	Hala Kondratowa	1240	N 49.25715° E 019.96276°	Upper montane range	*Gladiolo-Agrostietum*
10	Polana Kopieniec	1236	N 49.27299° E 020.01854°	Upper montane range	*Gladiolo-Agrostietum*
11	Przehyba	1133	N 49.46750° E 020.55543°	Upper montane range	*Plagiothecio-Piceetum*
12	Przełęcz Bukowska	1117	N 49.05225° E 022.77288°	Upper montane range	*Poo-Deschampsietum*
13	Przełęcz Krowiarki	1058	N 45.58989° E 019.58641°	Lower montane range	*Plagiothecio-Piceetum*
14	Kuźnice	1000	N 49.27168° E 019.98218°	Lower montane range	*Arrhenatheretum alatioris*
15	Dolina Kościeliska	980	N 49.26355° E 019.87252°	Lower montane range	*Gladiolo-Agrostietum*
16	Zakopane TPN	902	N 49.28448° E 019.97127°	Lower montane range	*Gladiolo-Agrostietum*
17	Hala Boracza	863	N 49.54587° E 019.16575°	Lower montane range	*Gladiolo-Agrostietum*
18	Brzegi Górne	773	N 49.14267° E 022.56499°	Lower montane range	*Gladiolo-Agrostietum*
19	Biała Woda	771	N 49.39515° E 020.59550°	Lower montane range	*Anthylii-Trifolietum montani*
20	Lubomierz	768	N 49.59272° E 020.22380°	Lower montane range	*Anthylii-Trifolietum montani*
21	Wołosate	761	N 49.06482° E 022.68687°	Lower montane range	*Gladiolo-Agrostietum*
22	Żabnica	736	N 49.55321° E 019.20479°	Lower montane range	*Gladiolo-Agrostietum*
23	Uhryń	713	N 49.47237° E 020.86013°	Lower montane range	*Gladiolo-Agrostietum*
24	Tarnawa	677	N 49.12730° E 022.80056°	Lower montane range	*Arrhenatheretum alatioris*
25	Kocoń	630	N 49.74408° E 019.40473°	Lower montane range	*Gladiolo-Agrostietum*
26	Ciechań	629	N 49.45070° E 021.49384°	Lower montane range	*Carlino-Dianthetum*
27	Kamesznica	626	N 49.60177° E 019.05208°	Lower montane range	*Gladiolo-Agrostietum*
28	Ludźmierz	605	N 49.47160° E 019.96879°	Lower montane range	*Arrhenatheretum alatioris*
29	Łopienka	592	N 49.26173° E 022.36151°	Lower montane range	*Gladiolo-Agrostietum*
30	Michniowiec	591	N 49.30973° E 022.72639°	Lower montane range	*Gladiolo-Agrostietum*
31	Sromowce Niżne	548	N 49.39893° E 020.39525°	Lower montane range	*Anthylii-Trifolietum montani*
32	Żydowskie	499	N 49.47831° E 021.46578°	Foothills	*Arrhenatheretum alatioris*
34	Gaboń	488	N 49.51427° E 020.55303°	Foothills	*Arrhenatheretum alatioris*
34	Kamienica	475	N 49.58584° E 020.32247°	Foothills	*Arrhenatheretum alatioris*
35	Kalnica	471	N 49.36662° E 022.19975°	Foothills	*Arrhenatheretum alatioris*
36	Nieznajowa	462	N 49.49251° E 021.39219°	Foothills	*Arrhenatheretum alatioris*
37	Serednica	454	N 49.50097° E 022.48839°	Foothills	*Arrhenatheretum alatioris*
38	Rytro	452	N 49.48491° E 020.69088°	Foothills	*Arrhenatheretum alatioris*
39	Rozstajne	452	N 49.48896° E 021.41798°	Foothills	*Arrhenatheretum medioeuropaeum*
40	Paszowa	439	N 49.54168° E 022.41953°	Foothills	*Arrhenatheretum medioeuropaeum*
41	Kąty	407	N 49.55333° E 021.51637°	Foothills	*Arrhenatheretum alatioris*
42	Stefkowa	406	N 49.46311° E 022.47645°	Foothills	*Arrhenatheretum medioeuropaeum*
43	Stasiówka	395	N 50.00342° E 021.46434°	Foothills	*Arrhenatheretum alatioris*
44	Krempna	386	N 49.51071° E 021.49821°	Foothills	*Arrhenatheretum alatioris*
45	Polichty	362	N 49.81676° E 020.87115°	Foothills	*Arrhenatheretum medioeuropaeum*
46	Zawada	354	N 49.98436° E 021.00930°	Foothills	*Arrhenatheretum medioeuropaeum*
47	Naszacowice	342	N 49.54670° E 020.56232°	Foothills	*Arrhenatheretum medioeuropaeum*
48	Gołkowice Dolne	317	N 49.54876° E 020.57999°	Foothills	*Arrhenatheretum medioeuropaeum*
49	Pleśna	263	N 49.92034° E 020.94001°	Foothills	*Arrhenatheretum medioeuropaeum*

**Table 2:** Percentage share and abundance of coprophagous hydrophilids (Coleoptera, Hydrophilidae, Sphaeridiinae) caught in baited traps in the Polish Carpathians in 2011–2013.

No	Species	N	%	Class of Domination
1	*Cercyon (Cercyon) impressus* (Sturm, 1807)	3688	38.46%	Superdominant
2	*Sphaeridium lunatum* Fabricius, 1792	2762	28.80%	Dominant
3	*Cercyon (Cercyon) lateralis* (Marsham, 1802)	1126	11.74%	
4	*Cercyon (Cercyon) castaneipennis* Vorst, 2009	780	8.13%	
5	*Sphaeridium scarabaeoides* (Linnaeus, 1758)	382	3.98%	Subdominant
6	*Cryptopleurum minutum* (Fabricius, 1775)	372	3.88%	
7	*Megasternum immaculatum* (Stephens, 1829)	118	1.23%	
8	*Sphaeridium bipustulatum* Fabricius, 1781	88	0.92%	Subrecedent
9	*Cercyon (Cercyon) pygmaeus* (Illiger, 1801)	72	0.75%	
10	*Cercyon (Cercyon) haemorrhoidalis* (Fabricius, 1775)	59	0.62%	
11	*Cercyon (Cercyon) tatricus* Endródy-Younga 1967	54	0.56%	
12	*Sphaeridium marginatum* Fabricius 1787	45	0.47%	
13	*Cercyon (Cercyon) melanocephalus* (Linnaeus, 1758)	19	0.20%	
14	*Cercyon (Cercyon) quisquilius* (Linnaeus, 1761)	13	0.14%	
15	*Pachysternum capense* (Mulsant, 1844)	6	0.06%	
16	*Cercyon (Cercyon) unipunctatus* (Linnaeus, 1758)	4	0.04%	
17	*Cercyon (Paracycreon) laminatus* Sharp, 1873	1	0.01%	
	Total	9589	100.00%	

**Table 3:** Coprophagous hydrophilid beetles recorded in 2011–2013 at sites in the Polish Carpathians.

No	Site	1	2	3	4	5	6	7	8	9	10	11	12	13	14	15	16	17	Number of Species at Site
1	**Kasprowy Wierch**	●		●	●	●	●				●	●		●	●				9
2	**Twarda Galeria**	●	●	●	●						●	●							6
3	**Kocioł Mułowy**	●			●	●					●	●			●				6
4	**Hala Gąsienicowa**	●			●		●	●			●	●							6
5	**Upłaziańska Kopa**	●	●	●	●	●	●			●	●	●		●	●	●			12
6	**Skupniów Upłaz**	●			●	●													3
7	**Tarnica**	●	●	●	●	●	●		●		●				●			●	10
8	**Połonina Caryńska**	●	●	●	●	●	●		●				●	●					9
9	**Hala Kondratowa**	●										●							2
10	**Polana Kopieniec**	●		●	●														3
11	**Przehyba**	●	●	●	●	●	●	●			●			●					9
12	**Przełęcz Bukowska**	●	●	●		●	●												5
13	**Przełęcz Krowiarki**		●																1
14	**Kuźnice**	●	●	●	●	●	●			●	●			●					9
15	**Zakopane**	●	●	●	●		●	●			●								7
16	**Dolina Kościeliska**	●	●	●															3
17	**Hala Boracza**	●	●	●	●	●	●	●		●					●				9
18	**Brzegi Górne**	●	●	●	●	●	●	●	●	●				●					10
19	**Biała Woda**	●		●										●					3
20	**Lubomierz**	●	●	●	●		●	●											6
21	**Wołosate**	●		●															2
22	**Żabnica**	●	●	●	●	●	●	●											7
23	**Uhryń**	●	●	●	●	●													5
24	**Tarnawa**	●	●	●				●											4
25	**Kocoń**	●		●	●		●			●	●								6
26	**Ciechań**	●	●	●	●	●	●		●	●	●		●	●	●	●	●		14
27	**Kamesznica**	●		●			●												3
28	**Ludźmierz**	●	●	●	●	●					●						●		7
29	**Łopienka**	●	●	●	●	●	●		●		●		●						9
30	**Michniowiec**		●	●	●	●													4
31	**Sromowce Niżne**	●	●	●		●		●			●								6
32	**Żydowskie**	●	●		●	●	●		●	●									7
33	**Gaboń**	●	●	●	●	●		●			●		●						8
34	**Kamienica**	●	●	●	●		●				●								6
35	**Kalnica**	●	●	●	●	●	●		●					●					8
36	**Nieznajowa**	●	●	●	●	●	●		●	●									8
37	**Serednica**	●	●	●	●	●			●				●						7
38	**Rytro**	●	●	●	●	●	●						●						7
39	**Rozstajne**	●	●	●	●	●	●		●	●			●			●			10
40	**Paszowa**	●	●	●	●	●			●										6
41	**Kąty**		●			●													2
42	**Stefkowa**	●	●	●		●	●		●				●						7
43	**Stasiówka**	●	●	●		●				●						●			6
44	**Krempna**	●	●	●	●	●	●	●		●			●						9
45	**Polichty**	●	●	●	●	●													5
46	**Zawada**			●		●	●						●			●			5
47	**Naszacowice**	●	●	●	●	●	●		●										7
48	**Gołkowice Dolne**	●	●	●	●	●	●	●	●	●	●		●		●		●		13
49	**Pleśna**	●	●	●	●	●			●				●						7

**Legend:** 1—*Cercyon impressus*; 2—*Sphaeridium lunatum*; 3—*Cercyon lateralis*; 4—*Cercyon castaneipennis*; 5—*Sphaeridium scarabaeoides*; 6—*Cryptopleurum minutum*; 7—*Megasternum immaculatum*; 8—*Sphaeridium bipustulatum*; 9—*Cercyon pygmaeus*; 10—*Cercyon haemorrhoidalis*; 11—*Cercyon tatricus*; 12—*Sphaeridium marginatum*; 13—*Cercyon melanocephalus*; 14—*Cercyon quisquilius*; 15—*Pachysternum capense*; 16—*Cercyon unipunctatus*; 17—*Cercyon laminatus*.
